# The Hidden Pandemic: Impact of the COVID-19 Pandemic on Trauma Cases Due to Domestic Violence Admitted to the Biggest Level-One Trauma Center in Austria

**DOI:** 10.3390/jcm13010246

**Published:** 2023-12-31

**Authors:** Rita Babeluk, Bernhard Maier, Timothée Bach, Stefan Hajdu, Manuela Jaindl, Anna Antoni

**Affiliations:** Division of Trauma Surgery, Department of Orthopedics and Trauma Surgery, Medical University of Vienna, Waehringer Gürtel 18-20, 1090 Vienna, Austria; bernhardmaier13@gmail.com (B.M.); timo@bach.at (T.B.);

**Keywords:** COVID-19, domestic violence, interpersonal violence, trauma

## Abstract

Background: An alarming increase in domestic violence was reported during the COVID-19 pandemic worldwide. The aim of this study is to investigate changes in the frequency and the nature of domestic violence at the largest level-one trauma center in Austria. Methods: All patients admitted to our institution with domestic violence injuries 15 months before and after the beginning of the COVID-19 pandemic were included. For our analysis, we investigated the frequency of trauma patients after domestic violence in relation to all other trauma patients. Furthermore, age, sex, citizenship, injury pattern, injured body regions, injury mechanism, offender–victim relationship, and hospitalization rate were also analyzed. Results: Among all trauma patients admitted, the ratio of patients who reported domestic violence injuries increased from 0.465% to 0.548% since the start of the pandemic. In addition, out of the total count of domestic violence victims, the percentage of Austrian citizens increased significantly from 51.2% to 60.6% (*p* = 0.016). All other parameters showed no significant changes pre and post-pandemic. Conclusion: The COVID-19 pandemic contributed to a relative increase in patients with domestic violence injuries at the largest trauma unit in Austria, along with a significant increase among Austrian citizens. The remaining study parameters did not differ significantly, indicating that the frequency changed during the pandemic but not the underlying pattern of domestic violence.

## 1. Introduction

In 2020, the COVID-19 pandemic quickly spread around the world and became one of the most important global health concerns [1]. To prevent health care systems from collapsing and flatten the hospitalization curve, governments around the world, including Austria, mandated social restrictions such as home offices, homeschooling, social distancing, stay-at-home orders, and other measures. Throughout the pandemic, the Austrian government established three lockdowns nationwide. The first nationwide lockdown began on 16 March 2020, with loosening restrictions coming into effect on 14 April 2020. A second lockdown was in place from 17 November 2020 to 7 December 2020. A third one was implemented from 26 December 2020 to 8 February 2021. Additionally, a fourth more targeted lockdown was put in place from 1 April 2021 to 5 May 2021 in the eastern part of Austria, including Vienna, the region of our investigation. Even between and after the lockdowns, many people worked from home and taught their children through homeschooling. The government advised against gatherings in larger groups, and large indoor events were canceled for a long time [2]. The pandemic not only challenged the healthcare system but also affected world economics and the financial stability of many people [3]. Studies have shown that stress, economic factors, emotional disappointment, inadequate housing, and substance abuse are triggers of violence within family units [4]. Thus, it comes as no surprise that the COVID-19 pandemic contributed to an increase in domestic violence globally, as demonstrated in previous studies [5,6,7,8,9,10]. Similarly to the health pandemic, a spike in domestic violence was observed in the aftermath of natural disasters [11], as was the case after Hurricane Katrina in the U.S. in 2005 [12] and also in 2004 after the tsunami in Sri Lanka [13]. In both of these cases, intimate partner violence increased significantly.

According to the UN, domestic violence is defined as a “pattern of behavior in any relationship that is used to gain or maintain power and control over an intimate partner”. This definition can further be expanded to include violence by ex-partners or family members [14]. Additionally, domestic violence can manifest via a complex pattern of behaviors that can include sexual and emotional abuse as well as physical acts of violence within the personal environment. Other forms of violence can play a role as well, such as the use of socioeconomic control and verbal abuse. A personal environment may include homes, workplaces, or other places of close proximity. Domestic violence may include one or more forms of violence [15]. Moreover, women are far more likely to be affected than men. Approximately 80% of domestic violence victims are female [16].

In Austria, a European country with currently 9.1 million inhabitants, more than a third of women (34.51%) between the ages of 18 and 74 are exposed to domestic violence at least once during their lifetime, with a high number of unreported cases noted [17]. According to crime statistics, 28 femicides were recorded in 2021 and 29 in 2022 [18]. Despite the alarming statistics, talking about domestic violence is often difficult for victims. Oftentimes, self-blaming, shame, social stigma, or the fear of being judged are a major hurdle for victims to seek help. Other factors, such as the fear of further escalation or economic dependence on a partner, are reasons often cited by victims for looking for support late. It is known that the more potent the threats against victims, the more severe the violence, and the stronger the dependency, the more challenging it becomes for individuals to actively seek help [19].

According to Austrian law, every hospital must have a team to protect individuals experiencing domestic or sexual violence. The team must include an interdisciplinary panel of psychiatrists, gynecologists, trauma surgeons, emergency medicine physicians, nurses, and psychologists. They closely collaborate with social workers and the police. The aim of the team is to assist medical professionals in counseling and supporting victims as well as to raise awareness to improve the early detection of physical, psychological, and sexual violence. The team is also a link between hospitals, women’s shelters, and other institutions. In 2008, the University Hospital of Vienna was one of the first hospitals in Austria to establish such an interdisciplinary team [20,21].

To our knowledge, trauma cases in Austria caused by domestic violence during the COVID-19 pandemic have not yet been investigated.

The aim of the following study is to investigate whether the COVID-19 pandemic, which was accompanied by social distancing and socioeconomic uncertainties, impacted the trauma cases of domestic violence at an urban level-one trauma center in Vienna. It is important to note that the level-one trauma center of Vienna is one of the biggest trauma centers in central Europe, treating 51.293 outpatients and 566 shock room patients per year (2021). This study not only investigates the number of cases but also focuses on the question of whether the underlying patterns of domestic violence changed during the pandemic.

## 2. Materials and Methods

A retrospective single-center study was performed. The medical records of all in- and outpatients who were admitted to the Division of Trauma Surgery, Department of Orthopedics and Trauma Surgery, Medical University of Vienna, between December 2018 and May 2021 and were registered by the victim protection group were reviewed for this study. Furthermore, only patients who reported injuries caused by domestic violence were included in the study. Patients with disputable third-party involvement or who were later found not to be injured by an act of domestic violence were excluded from the study, along with patients younger than 18 years. Finally, patients were numbered consecutively and pseudonymized.

Two time periods were compared with each other: 15 months before (PRE: December 2018–February 2020) and 15 months after (COVID-19: March 2020–May 2021) the outbreak of the pandemic in Austria. In an additional analysis, 12 comparable months (March 2019–February 2020 vs. March 2020–February 2021) in both periods were compared to exclude any seasonal variations not caused by the pandemic.

The following parameters were obtained from the medical records: age, sex, citizenship, trauma mechanism (e.g., blunt/sharp weapon, punching, and biting), time period between injury and doctor’s visit in our department, relationship of the offender and the victim, hospitalization rate, number of injured body regions, and injury types.

Eleven different injury types were predefined: (1) Contusion or distortion; (2) laceration wounds; (3) cuts or stab wounds; (4) fractures, tooth lesions, and dislocations; (5) Hematomas; (6) abrasions and degloving; (7) concussion; (8) intracerebral bleeding; (9) bite wounds; (10) internal organ injuries; (11) burns. To provide an index for the sum of injury types, an unweighted sum of categories was generated.

The number of injured body regions was summarized based on the recorded diagnoses to reflect the minimum amount of strikes or other physical acts of violence performed by the offender.

Furthermore, eight trauma mechanisms were predefined to classify the entire spectrum of injuries: (1) violence without a weapon (e.g., punching, kicking, and pushing); (2) use of a blunt weapon (e.g., rolling pin); (3) use of a sharp weapon (e.g., knife or broken glass); (4) strangling; (5) biting; (6) burning; (7) injury in the context of sexual violation; (8) no specified modality.

The documented citizenship of the patients in the medical records was grouped into geographic regions for statistical analysis. The ethnicity of the patients could not be determined at the time of the survey; therefore, their citizenship was used to evaluate possible demographic changes among the victims after the outbreak of the pandemic.

Data analysis was performed using IBM SPSS^®^ version 27.0. We employed descriptive statistics (means, standard deviations (SD), and/or percentages) to present the characteristics comparing the two time periods. The significance of metric data was determined using the Mann–Whitney U test. The chi-square test and Fisher’s exact test were applied for the between-group analysis for nominal data. The significance level for all analyses was set at *p* = 0.05.

The study was approved by the ethics committee of the Medical University of Vienna (ECS 2104/2021) and performed according to the guidelines of the Declaration of Helsinki (1964), including current revisions.

## 3. Results

In the 15 months preceding COVID-19, the average monthly number of trauma patients admitted to the emergency unit was 3559 (minimum 3119; maximum 4293). After the outbreak of the pandemic, between March 2020 and May 2021, the average number of patients in the emergency unit was 2347 per month (minimum 1333; maximum 3099), representing a 34.1% decrease in trauma patients after the beginning of the pandemic. The number of patients presenting injuries due to domestic violence averaged 17 (minimum 8; maximum 27) per month during the studied period before the pandemic and 13 (minimum 5; maximum 21) per month thereafter, representing a 22.2% decrease.

The proportion of patients in trauma surgery at the emergency unit who reported injuries in the context of domestic violence compared to all presenting trauma patients was 0.465% during the 15-month observation period before the outbreak of COVID-19. This number has increased to 0.548% in the 15 months after the beginning of the pandemic.

**Figure 1:**
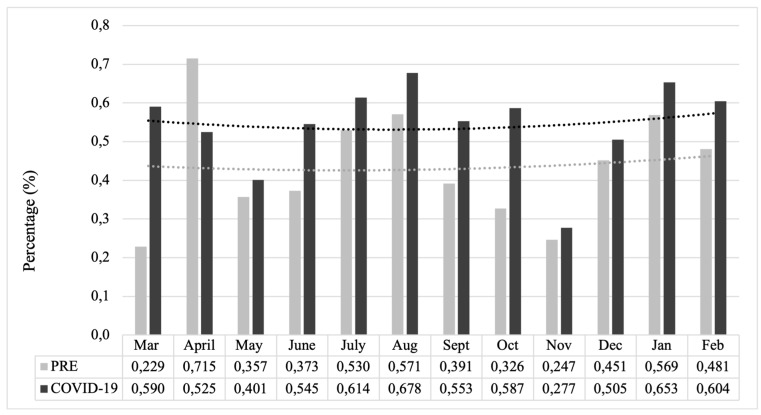
Relative number of domestic violence vs. all trauma cases represented with polynomial trend lines (dotted lines). Proportion value of domestic violence in relation to all trauma cases at the Division of Trauma Surgery of the Medical University of Vienna in comparison of the two observation periods represented with polynomial trend lines (PRE March 2019–February 2020 vs. COVID-19 March 2020–February 2021).

Relative frequencies of injuries caused by domestic violence compared to all trauma patients in the comparable 12 months of the two time periods, before and during COVID-19, increased from an average of 0.437% (minimum 0.229%; maximum 0.715%) up to 0.544% (minimum 0.277%; maximum 0.678%). The difference in the two trend lines indicates a relative increase in domestic violence cases during COVID-19 (Figure 1).

**Figure 2:**
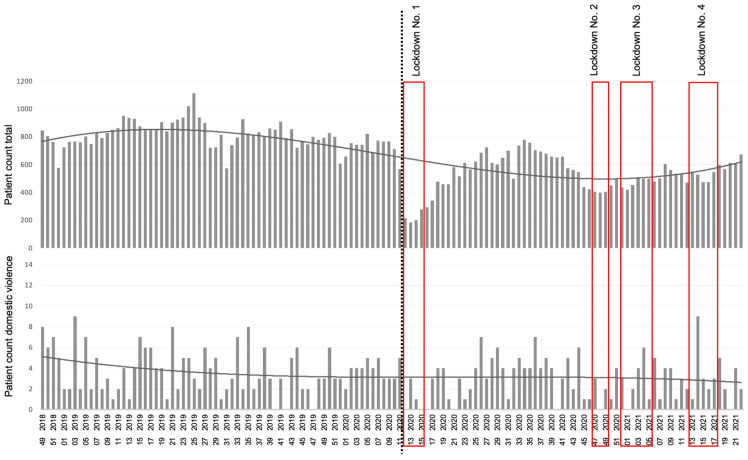
Weekly domestic violence vs. all trauma patients over the entire study period. Patient counts in total and with domestic violence injuries per week over the entire study period represented with polynomial trend lines. The dotted line marks the beginning of the pandemic; the periods of lockdown in Vienna are indicated in boxes. The x-axis shows the calendar week and the year above.

Figure 2 shows the weekly number of trauma patients and patients with injuries due to domestic violence at the Department of Trauma Surgery over the entire study period. The dotted line represents the start of the COVID-19 pandemic. The mandatory lockdowns in Vienna are shown in boxes. Especially in the first lockdown, the total number of trauma patients decreased markedly. In the following lockdowns, this effect was observed to a lesser degree. Overall, the trend lines show that the total number of trauma patients decreased after the start of the pandemic, while the number of injuries caused by domestic violence remained stable, resulting in a relative increase in domestic violence cases.

The proportion of female domestic violence patients before COVID-19 was 88.3% (219 cases), whereas this proportion reached 89.6% (173 cases) during the pandemic without statistical significance (*p* = 0.659). None of the patients in this study identified themselves as gender diverse.

The median age of domestic violence patients was 35.8 years and 33.9 years before and after the rise of COVID-19, showing no significant decrease, *p* = 0.406. The median age was not significantly different for women and men in either time period, PRE *p* = 0.165, COVID-19 *p* = 0.859. Considering the patients’ sex separately, there was a non-significant decrease in age for both females, from 35.2 to 34.0 years, *p* = 0.585, and males, from 40.5 to 31.5 years, *p* = 0.404.

The mean time between a domestic violence assault and admission at the trauma center showed no significant difference between the two observation periods, PRE 0.88 ± 3.21 days (min 0–max 22; *n* = 244) vs. COVID-19 0.61 ± 1.53 days (min 0–max 9; *n* = 191), *p* = 0.906. In six cases, the time of the assault was unknown.

The hospitalization rate also showed no significant distribution difference between the two observation periods: PRE 15.3% vs. COVID-19 14.5%, *p* = 0.812.

When injury patterns were compared between the two observation periods, no significant changes were observed. However, there was a significant increase in lacerating wounds observed during the pandemic. Multiple records were considered in this analysis since a patient could have more than one injury type. Table 1 compares the injury types in the two observation periods.

The number of injured body regions indicated a non-significant increase (*p* = 0.833) with a mean of 3.14 ± 2.20 injured regions pre-COVID-19 versus 3.19 ± 2.33 after the onset of the pandemic. The mean number of different injury types per patient also did not significantly rise from 1.37 to 1.48, *p* = 0.138.

The trauma mechanisms or the nature of the assault did not show any significant changes when comparing the two observation periods (Table 2). Multiple records were considered for this analysis.

There was no significant shift in the offender profile between the two observation periods. Before the onset of the pandemic, 53.6% of violent offenders were the partner of the victim and 23.0% the ex-partner. This changed to 56.0% and 17.6%, respectively, during the pandemic, *p* = 0.386.

The proportion of citizenships of the patients summarized by regions showed statistically significant changes when comparing the two observation periods, as shown in Table 3. Furthermore, Austrian citizens relatively increased.

## 4. Discussion

This study shows a relative increase in patients with domestic violence injuries after the outbreak of the COVID-19 pandemic. No changes in injury patterns were observed. The comparison of seasonal observation periods also showed a substantial increase in domestic violence during the pandemic. This increase is consistent with findings from previous studies [22,23,24,25,26,27]. In Austria, during the first year of the COVID-19 crisis, namely 2020, there was a significant increase in phone calls to the Women’s Helpline Against Violence. Women’s Helpline caseworkers took 71 percent more calls in March, April, and June 2020 and 33 percent more calls in December 2020 than the years prior [28].

The results of this study show that the total number of emergency unit visits decreased remarkably after the outbreak of the COVID-19 pandemic by 34.1% and even more drastically during the first lockdown. This correlates with studies from other countries showing that hospital visits during COVID-19 decreased drastically because patients were fearful of infection. This has been shown to even be the case for life-threatening conditions such as acute myocardial infarction or cancer [29,30,31]. Additionally, a decrease in trauma admissions such as traffic accidents, work-related accidents, and sports accidents was shown in several studies around the world [32,33,34,35]. However, it is evident that there has been a relative decrease in emergency unit visits for domestic violence patients compared to the total number of trauma cases. In fact, domestic violence patients’ visits to the emergency unit have decreased by 22.2%, while the total number of trauma cases has decreased by 34.1%. The fact that domestic violence cases have decreased less remarkably than the overall number could potentially indicate that domestic violence in Austria increased during the pandemic, assuming that the fear of acquiring COVID-19 in the hospital is equally distributed among all trauma victims.

To our knowledge, there has not been any study examining the change in the patterns of domestic violence before and after the onset of the pandemic. In this study, the sex ratio, age, offender profile, trauma mechanisms, injury type, and hospitalization rate showed no significant differences in the two time periods. The results indicate that only lacerations had a significantly higher proportion value (15.5%) during the COVID-19 pandemic, *p* = 0.045.

This study adopts a unique approach by utilizing the number of injured body regions as a metric to gauge the degree of aggression. This metric assumes that the extent of bodily harm suffered can serve as an indicator of the intensity and severity of the assault. For instance, if a patient exhibits injuries to the head, one arm, and the thorax, it implies that a minimum of three impacts occurred. This may suggest that a potentially higher degree of escalation has occurred compared to cases where only one body part is affected. This methodology is grounded in the assumption that the more body regions affected, the more intense and aggressive the assault may have been. The choice of this metric reflects an attempt to quantify and characterize the level of violence or force exerted on the victim during a specific incident. In this study, no significant changes in the number of injured body regions could be detected between the two study periods.

This study shows a significant shift in the geographic pattern regarding the citizenship of the patients. There was a relative increase in Austrian citizens who were victims of domestic violence from 51.2% to 60.6%. One explanation for the increase could be the limitations on international travel during the pandemic. The barriers to transnational mobility caused by the pandemic also led to workforce shortages in certain sectors, such as seasonal workers in agriculture and tourism in Austria [36]. In addition, the massive decrease in migrant workers after the start of the pandemic could also explain the significant increase in domestic violence among Austrian citizens.

In this study, all patients identified themselves as either female or male. No one identified as diverse, transgender, or nonbinary. Therefore, the number of gender-diverse patients in this study is not known. However, it is known that members of the transgender and gender-diverse community are much more likely to experience domestic violence and sexual violence. Further studies are required to shed more light on this aspect [37,38].

Domestic violence is influenced and enhanced by various factors such as legal, political, cultural, and economic dynamics [39], which has led to a “hidden pandemic” as part of the COVID-19 crisis. It was previously documented that crises such as the Great Recession in the United States [40] or the European debt crisis in 2010 [41] led to an increase in domestic violence due to increasingly stressful environments and economic instability [42,43,44]. The fact that the increase in domestic violence did not occur on a massive scale in Austria as seen in other countries may be due to the fact that a large-scale social program was put in place early on to mitigate the socioeconomic consequences of the health and social crisis that ensued. Some of these programs included financial support for small and medium-sized enterprises, the tourism sector, reduced working hours programs, and other governmental benefits [45]. On the other hand, when it comes to femicides, Austria fared worse than other European countries. Austria is the only country in the European Union where more women compared to men are murdered [46]. In 2018 and 2019, a total of 41 and 39 femicides were documented in Austria, respectively. During COVID-19, the number of femicides decreased slightly to 31, 28, and 29 in the years 2020, 2021, and 2022, respectively [28]. In an international context, these numbers are alarmingly high for a small country like Austria, with a population of approximately nine million. Comparatively, Germany, a neighboring country, which is 10 times the size of Austria, registered 181 femicides in 2022, represents 1 femicide per 364,190.88 inhabitants in Austria versus 1 femicide per 870,948.47 inhabitants in Germany [47,48].

Medical facilities are often the first point of contact for people seeking help after domestic violence assault. Further statistics show that 60% of women affected by interpersonal violence first seek help in medical facilities, while 23% of them seek help in hospitals [49]. In those cases, patients find themselves in emotionally challenging situations. The victims are often physically and psychologically traumatized. They often feel shame and carry self-blame, and are afraid of stigmatization in addition to the fear that violence may further escalate from their offenders. Most of the time, these victims are socially or financially dependent on the offenders, especially after a longer history of violence has occurred within the family. That all makes it often difficult for victims to speak up and seek help [19]. This situation underlines the importance of awareness and knowledge of local victim protection facilities among healthcare professionals to initiate help for victims of domestic violence.

Domestic violence is a topic that has methodological limitations due to its context. The number of unreported cases of domestic violence is not reflected in this study. It is not known how it has changed in the times of COVID-19. It should also be noted that violence in the form of psychological and verbal abuse is not included in this study. Another limitation of this study is its retrospective nature. However, the question remains whether the number of patients would have differed had the study been carried out in a prospective setting.

Interdisciplinary teams are impactful instruments to help detect domestic violence incidents, protect victims, and subsequently prevent further escalations. Even though all hospitals in Austria are equipped with such teams to assist and enhance support for victims, additional action programs are certainly required to further minimize domestic violence, especially in times of crisis.

## 5. Conclusions

Domestic violence is an omnipresent societal challenge, transcending age, gender, ethnicity, and socioeconomic status. It is a disturbing phenomenon that jeopardizes not only the physical well-being of victims but also inflicts lasting psychological trauma. This issue has proven to be resilient, persisting across diverse demographics and reinforcing the notion that it is not limited to any particular group [50].

The study at hand sheds light on a troubling aspect of domestic violence, its tendency to escalate during periods of crisis, as observed during the COVID-19 pandemic and when compared to the overall trauma patient population. Despite the surge in the relative frequency of domestic violent assaults, the study reveals a consistency in underlying patterns. Factors such as age, gender, offender profile, time from assault to admission, and injury patterns did not exhibit significant changes, implying that while the frequency seems heightened, the fundamental characteristics of these incidents remained largely unaltered.

Primary care units, particularly trauma surgery departments, emerge as frontline responders in dealing with the aftermath of interpersonal violence. These units serve as the initial point of contact for individuals seeking assistance after experiencing domestic violence. A sensitive and empathetic approach from healthcare professionals becomes paramount in these settings. Equally crucial is the meticulous documentation of injuries, providing a foundation for legal and medical intervention and ensuring that the full extent of the harm is acknowledged.

In recognizing the role of primary care units, it becomes evident that they play a pivotal role not only in immediate medical care but also in offering essential information about available resources and options for individuals navigating the complex aftermath of domestic violence.

Domestic violence is a deep-rooted societal issue that demands comprehensive and sustained efforts. Combating this problem requires collaboration across various sectors, including healthcare, law enforcement, social services, and community support. It is imperative to raise awareness, foster sensitivity, and implement effective intervention strategies to provide a supportive environment for survivors and work towards the prevention of domestic violence in the first place, especially in times of crisis.

## Figures and Tables

**Table 1:** Total numbers and proportions of injury types considering the two observed time periods; multiple records were considered.

Injury Type	PRE	COVID-19	*p*-Value
contusion, distortion	211 (85.1%)	162 (83.9%)	0.742
lacerating wounds	23 (9.3%)	30 (15.5%)	0.045 *
cuts, stab wounds	12 (4.8%)	5 (2.6%)	0.224
fractures, tooth lesions, dislocation	29 (11.7%)	28 (14.5%)	0.382
hematomas	22 (8.9%)	24 (12.4%)	0.224
abrasions, degloving	33 (13.3%)	28 (14.5%)	0.717
concussion	6 (2.4%)	5 (2.6%)	>0.999
intracerebral bleeding	1 (0.4%)	0	>0.999
bite wounds	3 (1.2%)	1 (0.5%)	0.635
internal organ injuries	0	2 (1.0%)	0.191
burns	0	1 (0.5%)	0.438

* *p* ≤ 0.05.

**Table 2:** Total numbers and proportions of trauma mechanisms of assault considering the two observation periods; multiple records were considered.

Trauma Mechanism	PRE	COVID-19	*p*-Value
punching, kicking, pushing	227 (91.5%)	172 (89.1%)	0.392
blunt weapon	13 (5.2%)	13 (6.7%)	0.509
sharp weapon	11 (4.4%)	5 (2.6%)	0.304
strangling	6 (2.4%)	2 (1.0%)	0.475
biting	2 (0.8%)	2 (1.0%)	>0.999
burning	1 (0.4%)	0	>0.999
injury in context of a sexual violation	1 (0.4%)	0	>0.999
no specified modality	1 (0.4%)	1 (0.5%)	>0.999

**Table 3:** Total numbers and proportions of patient’s citizenships summarized by regions in the two observation periods.

Citizenship (Region)	PRE (n = 248)	COVID-19 (n = 193)	*p*-Value
Austria	127 (51.2%)	117 (60.6%)	0.016 *
Eastern Europe	69 (27.8%)	40 (20.7%)	
Southern Europe	7 (2.8%)	4 (2.1%)	
other European countries	8 (3.2%)	2 (1.0%)	
Africa	7 (2.8%)	1 (0.5%)	
Asia	8 (3.2%)	6 (3.1%)	
America	0	5 (2.6%)	
Middle eastern countries	18 (7.3%)	15 (7.8%)	
stateless	4 (1.6%)	1 (0.5%)	
not specified	0	2 (1.0%)	

* *p* ≤ 0.05.

## Data Availability

The data used and analyzed are available from the corresponding author solely upon reasonable request.
